# Prevalence of furcation‐involved molars in a Swedish adult population. A radiographic epidemiological study

**DOI:** 10.1002/cre2.27

**Published:** 2016-09-29

**Authors:** Uday Najim, Christer Slotte, Ola Norderyd

**Affiliations:** ^1^ Department of Periodontology The Institute for Postgraduate Dental Education Jönköping Sweden; ^2^ Department of Biomaterials, Institute for Clinical Sciences Göteborg University Göteborg Sweden; ^3^ Faculty of Odontology Malmö University Malmö Sweden

**Keywords:** Age, education level, gender, gingivitis, periodontal pockets, periodontitis, plaque, smoking, tooth furcation

## Abstract

The purpose of this study was to identify the prevalence of molars with furcation involvements grades II and III in adults participating in the Jönköping Oral Health Study 2003. The second aim was to study correlations between different variables and the presence of furcation involvement in these individuals. The present study was performed using bitewing and apical radiographs from 329 subjects. Furcations were considered healthy if the furcation was filled with bone up to the fornix. Two thousand fourteen molars fulfilled the inclusion criteria. The prevalence of molars with furcation involvements was 8.3%. Univariate analysis showed that plaque, age, and presence of periodontal pockets were significantly correlated with furcation‐involved molar/s (*P* ˂ 0.0001). Gingivitis and education were also significantly correlated to the presence of furcation involvement (*P* ˂ 0.006) and (*P* ≤ 0.01), respectively. Gender had no association with presence of involvements. Multivariate analysis showed that age and presence of periodontal pockets were significantly correlated with furcation involvement (*P* ˂ 0.0001). Smoking was also found to be associated with furcation involvement (*P* ˂ 0.04). The tooth most frequently and least likely displaying furcation involvement was the maxillary first molar and the mandibular second molar, respectively. Periodontal pockets, age, and smoking were risk indicators for furcation involvement.

## Introduction

The most frequently lost teeth in a population are the first and second molars (Corraini et al. 2008). Furcation involvements are common in patients with periodontitis (Svardstrom & Wennstrom 1996). The fate of molars is determined by the following factors: grade of furcation involvement, bone level, and smoking (Dannewitz et al. 2006, Svradstrom & Wennstrom 2000). Furcation involvement is frequently more common in maxillary molars than in mandibular molars (Ross & Thompson 1980, Svardstrom & Wennstrom 1996, Dannewitz et al. 2006). This can be explained by the difficulty in accessing the proximal surfaces on maxillary molars for cleaning (Ross & Thompson 1980, Dannewitz et al. 2006). A study by Ross and Thompson (1980) found that the prevalence of furcation involvement in maxillary molars was 90%; compared with 35% in mandibular molars. Studies on dry skulls have found that maxillary first and second molars have a higher risk for furcation involvement than mandibular molars. Moreover, first molars were more frequently affected than second molars (Larato 1970, Tal & Lemmer 1982). Radiographs have been used to determine the presence or absence of furcation involvements with different results (Rees et al. 1971, Deas et al. 2006). Rees et al. found that 86% of the buccal and lingual furcation can be diagnosed with the aid of radiographs (Deas et al. 2006). Deas et al. found that the agreement on detection of approximal furcation involvement between clinical and radiographic examination was 38.7%. Furthermore, the agreement on the absence of furcation involvement was 92.2%.

Periodontitis is a multifactorial disease, that is, many factors participate in the initiation and progression of the disease (Albandar & Rams 2002, Albandar 2002, Nunn 2003). Some of these are smoking, gender, level of education, age, genetics, plaque, and gingival inflammation (Albandar 2005). Plaque is the main factor initiating the inflammatory reaction in periodontal tissue (Albandar 2005). Smoking is the main identifiable risk factor for chronic periodontitis (van Winkelhoff et al. 2001, Bergstrom 2004). Several studies have shown that age is strongly associated with bone and probing attachment loss (Papapanou et al. 1988, Papapanou et al. 1991, Gamonal et al. 1998, Albandar et al. 1999, Neely et al. 2001, Edman et al. 2012). An epidemiological study carried out in the USA showed that pocket depth, attachment level, and molars with furcation involvement increased with age (Albandar et al. 1999). Education and socioeconomic status have a considerable impact on the periodontal status (Neely et al. 2001, Borrell et al. 2002). The prevalence of periodontitis has been found to be significantly higher in men than in women (Gamonal et al. 1998, Borrell et al. 2002). Attachment loss and furcation involvement have also been found to be considerably higher in men (Albandar et al. 1999). Gingival bleeding has been found to have a significant association with the development and progression of periodontitis (Albandar et al. 1998, Neely et al. 2001, Hyman & Reid 2003, Schatzle et al. 2003). A retrospective study, at a specialist clinic for treatment of periodontitis, showed that molars with grades II and III furcation involvement had about three and seven times higher risk, respectively, for progression of periodontitis and tooth loss (Salvi et al. 2014). Another study showed that furcations‐involved molars had higher risk for tooth loss than molars without furcation involvement (Hirschfeld & Wasserman 1978).

The aim of this radiological study was to evaluate the prevalence of furcation involvement in maxillary and mandibular molars in a Swedish adult population (the subjects were 40, 50, 60, and 70 years old).

A second aim was to study the correlation between different variables (gingivitis, smoking, plaque, level of education, presence of periodontal pockets, gender, and age) and the presence of furcation involvements.

## Material and Methods

This study was performed using the material from the Jönköping study 2003 and included individuals who lived in Jönköping City, Sweden. All individuals (*n* = 329), aged 40, 50, 60, and 70 years, were included in this study (Hugoson et al. 2005). The material of the original study was selected randomly from the register of the County Government Board. The total number of the participants was 987 individuals in the age group 3, 5, 10, 15, 15, 20, 30, 40, 50, 60, 70, and 80 years. The Jönköping epidemiological studies on oral health and disease started in 1973 and have been repeated every 10 years since then. The radiographs that were assessed in the current study were either panorals or periapicals or bitewings. These were examined by the author U. N. The radiographs were placed on an illuminated screen (Örebro, Sweden) with diffuse white light and then analyzed in a dark room with the aid of observation binoculars (Mattsson 1953)

Inclusion criteria:

1. All molars except wisdom teeth.
2. Individuals in the age groups 40, 50, 60, and 70 years.
3. Furcation involvement grades II and III (Hamp et al. 1975).

Exclusion criteria:

1. Untreatable molars and roots remnants have been considered as missing.
2. Dental implants have also been excluded.

Panorama was used to determine remaining and missing teeth. Bitewing radiographs were used to identify the furcation involvement.

The identification of furcation involvement was carried out according to this rule:

- The buccal furcation was considered healthy if the furcation fornix was filled with bone, or a black point in the furcation was seen, or a slight widening of the periodontal ligament in the furcation was confirmed, or if the alveolar bone crest was seen above or at the same level as the furcation fornix (the imaginary line between the mesial and distal alveolar bone crests was seen above or at the same level where the furcation fornix is confirmed). The furcation was considered involved if a radiolucent area was identified at the furcation fornix.
- The approximal furcations (mesial and distal) were considered healthy if the interdental alveolar bone crest was seen above or at the same level as furcation.

To ensure an adequate validity of the identifying of the furcation involvement, author U. N. examined 18 digital full mouth x‐ray sets of his own patients that had been referred to the Department of Periodontology at the Institute for Postgraduate Dental Education in Jönköping for treatment of periodontitis. This process took place before the commencement of the study. These patients were examined clinically and then full mouth radiographs were performed at the radiological department at the same institute. The sensitivity and specificity of identifying furcation involvement was 76.3% and 87.7%, respectively.

To ensure an acceptable interexaminer agreement of the diagnosis of furcation involvement a double measurement by the authors U. N. and O. N., was performed on 16 randomly chosen sets of radiographs from patients that belonged to the Jönköping study 2003. The Kappa‐value between the two observers was 0.88.

To ensure satisfactory intraexaminer accuracy, the author examined the 16 sets of radiographs twice with an interval of 2 weeks. The kappa‐agreement between the two observations regarding furcation involvement was 0.71. These measurements were performed before starting the study.

In order to study the relationship between clinical variables and furcation involvement, the following variables were used:

Plaque index: the presence of visible plaque was recorded for all tooth surfaces after drying with air according to the criteria for Plaque Indices 2 and 3 (Silness & Loe 1964).

Gingival index: the occurrence of gingival inflammation corresponding to Gingival Indices 2 and 3 was recorded for all tooth surfaces. Gingival inflammation was recorded if the gingiva bled on gentle probing (Loe & Silness 1963).

Smoking status: either current smoker or never/former smoker.

Presence of periodontal pockets: depth ≥ 4 mm (yes or no) on patient level.

Level of education: primary school/high school or college/university.

Gender: male or female. Age 40, 50, 60 and 70 years.

### Ethical consideration

The original study protocol was reviewed and approved by the research Ethical Committee at University of Linköping, Linköping, Sweden (Hugoson et al, 2005).

### Statistical analysis

The analysis was carried out at both univariate and multivariate level with spss. The questionnaires and clinical records were developed as a web survey in the software program esmaker nx2 (Entergate AB, Halmstad, Sweden). Frequencies, mean values, and distributions were calculated. Data processing was performed using spss version 22 (IBM Corporation, Armonk, NY).

## Results

**Figure 1:**
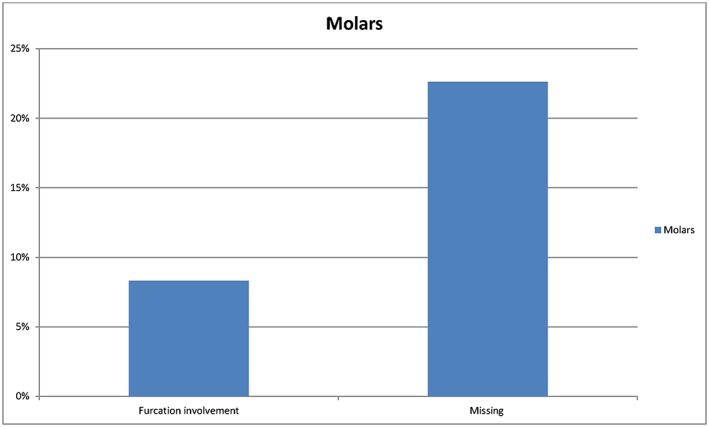
The prevalence of missing and furcation involvement at molars.

**Figure 2:**
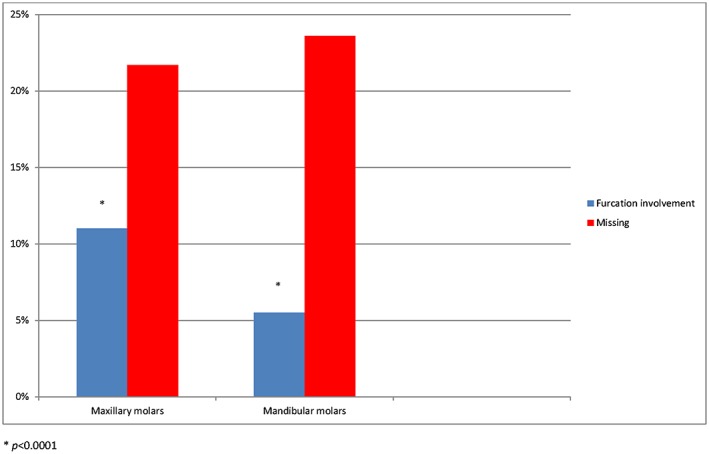
The prevalence of missing and furcation involvement at maxillary and mandibular molars.

**Figure 3:**
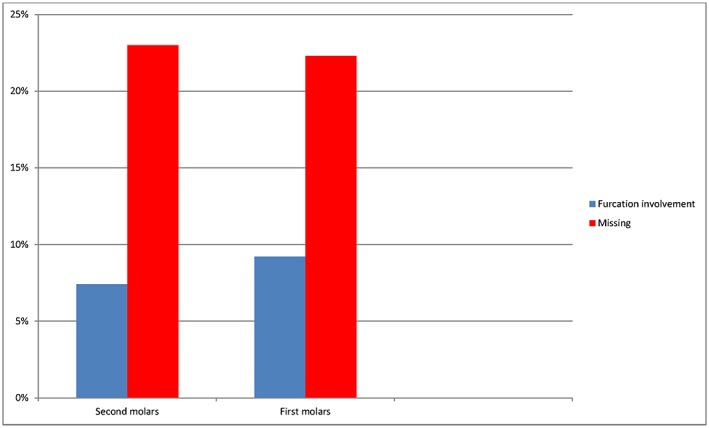
The prevalence of missing and furcation involvement at first and second molars.

**Figure 4:**
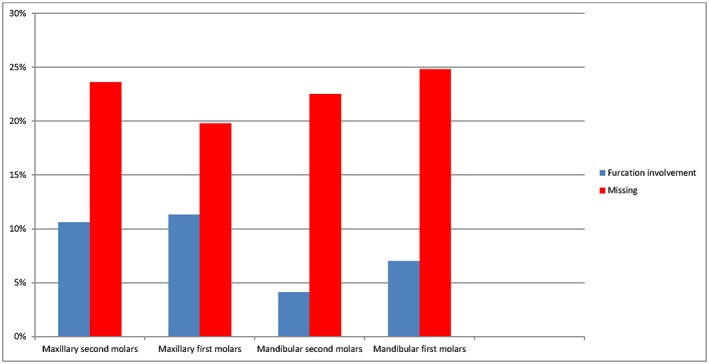
The prevalence of missing and furcation involvement at each molar.

Three hundred twenty‐nine individuals, 165 male and 164 female, aged between 40–70 years old with 2014 molars were included in the study. Of the 2014 molars, 1020 were in the maxilla and 994 were in the mandible. Figure 1 describes the prevalence of furcation‐involved molars, 167 from 2014 (8.3%), regarding grades II and III. Figure 2 shows the frequency of furcation involvement in the maxillary and mandibular molars, which was 112 from 1020 (11%) and 55 from 994 (5.5%), respectively. The greater number of maxillary molars displaying furcation involvement was statistically significant (*P* ˂ 0.0001). The frequency of missing maxillary and mandibular molars was 285 from 1316 (21.7%) and 331 from 1316 (23.6%), respectively. The difference in frequency of missing molars between the maxillary and mandibular molars was not significant. The first molars, 93 from 1009 (9.2%), had a higher frequency of furcation involvement than the second molars, which was 74 from 1005 (7.4%), Figure 3. The tooth that most frequently displayed furcation involvement was the maxillary first molar with 59 from 522 (11.3%), while the mandibular second molar was the least frequent 21 from 507 (4.1%), Figure 4. Twelve molars with root resection were documented in the population and were excluded from the results. Eight molars could not be examined because the radiographs were blurred.

Table 1 demonstrates the association between clinical variables and furcation involvement at univariate level. The level of education was significantly correlated with furcation involvement (*P* ≤ 0.01). Individuals with a low level of education had a higher risk of having molar/s with furcation involvement. Plaque, age, and presence of periodontal pockets were significantly correlated to furcation‐involved molar/s (*P* ˂ 0.0001). Gingivitis was significantly correlated to the presence of furcation involvement (*P* ˂ 0.006). Gender had no association to the outcome (*P* = 0.54).

**Table 1:** Clinical variables and furcation involvement (univariate logistic regression analyses).

Variable		*P*‐value		Odds ratio		95% C.I.
Education level		0.012		1.97		1.16–3.35
Periodontal pockets		0.0001		6.72		3.93–11.47
Gingival index		0.006		1.02		1.01–1.03
Plaque index		0.0001		1.02		1.01–1.03
Age		0.0001		1.06		1.03–1.08
Smoking		0.003		2.51		1.36–4.65
Sex		0.54		1.17		0.72–1.90

Multivariate analysis showed that the correlation between furcation involvement and age and the presence of periodontal pockets was statistically significant (*P* ˂ 0.0001). Smoking was also associated with furcation involvement (*P* ˂ 0.04). Level of education, plaque, gingivitis, and gender were not related to the outcome (Table 2).

**Table 2:** Clinical variables and furcation involvement (multivariate logistic regression analyses).

Variable		*P*‐value		Odds ratio		95% C.I.
Periodontal pockets		0.0001		5.00		2.76–8.70
Age		0.0001		1.06		1.03–1.09
Smoking		0.04		2.17		1.02–4.60

## Discussion

To our knowledge, this is the first radiological study of furcation involvement in a randomly selected population with different levels of periodontal health and disease experience. Larato studied the prevalence of molars with furcation involvement in 305 dry adult skulls. He found that the maxillary first molar was the tooth that most frequently displayed furcation involvement and mandibular second molars were the least frequent. The probability of having furcation involvement in the maxillary molars was higher than in the mandibular molars. These results are in agreement with the outcome presented in our study. The strength of Larato's study was that only skulls with little or no caries were included in order to minimize the risk of teeth having been extracted due to caries or pulpal indication. It is unclear when the individuals included in the study died, but as the study was carried out in 1970, it is likely that these individuals did not have access to dental care. This may affect the prevalence of furcation involvement; however, the pattern of the disease per se will not be affected. The author did not describe the prevalence of missing teeth, which may in turn influence distribution of the prevalence (Larato 1970). Tal studied the severity of furcation involvement in dry mandibles. This study consisted of 100 mandibles from individuals aged between 20–70 years. First molars were more affected regarding grades II and III involvement than the second molars (Tal & Lemmer 1982). This result is also in agreement with the outcome of our study. Svardstrom and Wennstrom (1996) studied the prevalence of furcation involvement in 222 patients who had been referred for treatment of periodontitis. The age group was 14–73 years old with 1570 molars. The prevalence of furcation‐involved molars was higher in the maxilla than in the mandible (Svardstrom & Wennstrom 1996). Despite the material being composed of individuals with periodontal disease, the results appear to be in agreement with our study.

Albandar et al. (1998) studied the effect of gingival inflammation and calculus on the progression of attachment loss. The individuals were examined 6 years after baseline. He found that teeth with gingival inflammation showed significantly higher attachment loss than teeth without inflammation (Albandar et al. 1998). This is confirmed in other longitudinal epidemiological studies (Neely et al. 2001, Schatzle et al. 2003). Over time, attachment loss in the furcation sites can eventually lead to furcation involvement. In other words, gingival inflammation might increase the risk of furcation involvement. This assumption is confirmed by our study. In a longitudinal epidemiological study, Schatzle et al. (2003) found that the quantity of plaque is associated with the degree of gingival inflammation. Increasing attachment loss was proportionally associated with gingival index and this association was significant, whereas plaque per se was not significantly associated with attachment loss. The fact that plaque does not affect the progression of periodontal disease was also confirmed in the studies carried out in Sri Lanka (Loe et al. 1986, Neely et al. 2001). Thus, the higher quantity of plaque is associated with higher scores on the gingival index and may indirectly eventually lead to an increased risk of furcation involvement. This is also confirmed by our study.

Albandar found that the frequency of furcation involvement is significantly higher in men than in women (Albandar et al. 1999). Several studies have shown that men have considerably deeper pockets and more attachment loss (Gamonal et al. 1998, Albandar et al. 1999). Salvi showed that the risk for loss of molars in patients treated for periodontitis was equally high in men and women (Salvi et al. 2014). In our study, we did not find any difference between men and women regarding furcation involvement. This may be because men and women in our population have a similar attitude towards dental health care (Norderyd et al. 1999). Several Swedish epidemiological studies have shown no difference between the genders regarding marginal bone loss and periodontal pockets ≥6 mm (Norderyd & Hugoson 1998, Wahlin et al. 2013, Jansson et al. 2014). Furthermore, Scandinavia has a long tradition of preventive dental care (Axelsson et al. 1991, Hugoson et al. 1998, Skudutyte‐Rysstad et al. 2007).

Papapanou studied bone level in individuals that were referred to the department of radiology for various reasons. He found that marginal bone loss increased with age and was more frequent around the maxillary teeth than the mandibular teeth (Papapanou et al. 1988). Okamoto found that periodontal pockets and attachment level around molars increase significantly with age in individuals with different periodontal disease status (Okamoto et al. 1988). Albandar found that the frequency of furcation involvement increased with age (Albandar et al. 1999). This was also confirmed in skull studies (Larato 1970, Tal & Lemmer 1982), which is in agreement with the outcome of our radiological study.

Gamonal found that only individuals with a high educational level had a healthy periodontal status. The prevalence of periodontal pockets (≥4 mm) was significantly higher in the group with high or basic schooling or without education than in individuals with a university education (Gamonal et al. 1998). Paulander found that individuals with a low education level were significantly overrepresented in the group with a higher value of mean attachment loss (Paulander et al. 2004). Because the likelihood of deeper pockets is associated with the level of education, the probability of having pockets at the furcation sites might be increased and might eventually influence the risk of furcation involvement. This assumption is supported by the outcome of the present study.

Haas found, in an epidemiological study with a 5‐year follow‐up, that smoking was a risk factor regarding attachment loss (Haas et al. 2014). Another epidemiological study showed that prevalence of moderate/severe periodontitis was significantly higher in smokers than in non‐smokers. Moreover, the percentage of teeth with attachment loss ≥5 mm was significantly higher in smokers (Albandar et al. 2000). Loss of attachment at the furcation may in turn lead to an increased risk of furcation involvement. This can also be confirmed by our study.

A weakness of the present study is that diagnosis of furcation involvement is a clinical variable. However, it should be noted that the agreement regarding the validity of the method used was high. Furthermore, the interexaminer and intraexaminer agreement was good.

To summarize, the prevalence of furcation involvement is significantly higher in maxillary molars than mandibular molars. Age, smoking, and presence of periodontal pockets are significantly associated with increased risk of furcation involvement.

The aim of this study was to study the frequency of furcation‐involved molars in a population with different levels of periodontal health and disease, that is, a population representative of Swedish society in general. This will highlight the need for different methods in order to optimize dental services by focusing resources on individuals with dental needs. Another reason behind the study is the fact that the involvement of furcation has a negative impact on the prognosis of the molars (Hirschfeld & Wasserman 1978, Salvi et al. 2014). Thus, it is important to establish the real prevalence. Unfortunately, we did not find any epidemiological studies that focused on furcation involvement and clinical variables (smoking, plaque, education level, and gingival index) despite the importance of furcation involvement to the prognosis of molars. This study might demonstrate the importance of including the diagnosis of furcation in future epidemiological studies.

## Clinical Relevance

Scientific rationale for the study: The aim of this project was to study the frequency of furcation‐involved molars in a population with different levels of periodontal disease experience, that is, a population representative of Swedish society in general.

Principle findings: The frequency of furcation involvement was significantly higher in the maxillary than mandibular molars. The tooth most frequently displaying furcation involvement was the maxillary first molar, while the mandibular second molar was the least likely to display furcation involvement. Multivariate analysis showed that the presence of periodontal pockets, age, and smoking was significantly associated with furcation involvement. Level of education, plaque, gingivitis, and gender were not related to the outcome.

Practical implications: Focusing dental resources on individuals with dental needs. Risk factors should be considered when a decision is taken regarding the treatment and maintenance of molars with furcation involvement.

## Conflict of Interest

The authors declare that they have no conflict of interests.

## Funding information

This study was supported by the Futurum Academy for Health and Care, Region Jönköping County.
